# Optimized cDNA libraries for virus-induced gene silencing (VIGS) using tobacco rattle virus

**DOI:** 10.1186/1746-4811-4-5

**Published:** 2008-01-22

**Authors:** Enwu Liu, Jonathan E Page

**Affiliations:** 1 NRC Plant Biotechnology Institute, 110 Gymnasium Place, Saskatoon, SK, S7N 0W9 Canada

## Abstract

**Background:**

Virus-induced gene silencing (VIGS) has emerged as a method for performing rapid loss-of-function experiments in plants. Despite its expanding use, the effect of host gene insert length and other properties on silencing efficiency have not been systematically tested. In this study, we probed the optimal properties of cDNA fragments of the *phytoene desaturase *(*PDS*) gene for efficient VIGS in *Nicotiana benthamiana *using tobacco rattle virus (TRV).

**Results:**

*NbPDS *inserts of between 192 bp and 1304 bp led to efficient silencing as determined by analysis of leaf chlorophyll a levels. The region of the *NbPDS *cDNA used for silencing had a small effect on silencing efficiency with 5' and 3' located inserts performing more poorly than those from the middle. Silencing efficiency was reduced by the inclusion of a 24 bp poly(A) or poly(G) homopolymeric region. We developed a method for constructing cDNA libraries for use as a source of VIGS-ready constructs. Library construction involved the synthesis of cDNA on a solid phase support, digestion with RsaI to yield short cDNA fragments lacking poly(A) tails and suppression subtractive hybridization to enrich for differentially expressed transcripts. We constructed two cDNA libraries from methyl-jasmonate treated *N. benthamiana *roots and obtained 2948 ESTs. Thirty percent of the cDNA inserts were 401–500 bp in length and 99.5% lacked poly(A) tails. To test the efficiency of constructs derived from the VIGS-cDNA libraries, we silenced the nicotine biosynthetic enzyme, putrescine *N*-methyltransferase (*PMT*), with ten different VIGS-*NbPMT *constructs ranging from 122 bp to 517 bp. Leaf nicotine levels were reduced by more than 90% in all plants infected with the *NbPMT *constructs.

**Conclusion:**

Based on the silencing of *NbPDS *and *NbPMT*, we suggest the following design guidelines for constructs in TRV vectors: (1) Insert lengths should be in the range of ~200 bp to ~1300 bp, (2) they should be positioned in the middle of the cDNA and (3) homopolymeric regions (i.e. poly(A/T) tails) should not be included. Our VIGS-cDNA library method, which incorporates these guidelines to produce sequenced, VIGS-ready cDNAs, will be useful for both fast-forward and reverse genetics experiments in TRV vectors.

## Background

Virus-induced gene silencing (VIGS) is a functional genomics tool that is increasingly used as an alternative to stable transformation-based RNA interference (RNAi) experiments in plants. Plants defend themselves against virus infection by targeting the viral genome for sequence-specific degradation [1-3]. This antiviral response is triggered by the presence of double-stranded RNA (dsRNA), which may occur as an intermediate in viral replication [4] or in highly-structured single-stranded RNA (ssRNA) viruses [5,6]. VIGS exploits the RNA silencing process by infecting plants with recombinant viruses containing host genes inserted in the viral genome, which results in the generation of small interfering RNAs (siRNAs) targeted against the corresponding host mRNAs. In effect, VIGS deceives a plant into identifying its own transcripts as viral RNA. Plant mRNAs targeted in this manner are degraded leading to a knockout or knockdown phenotype for the gene of interest.

Although transient in nature, and with its application limited by viral host range, VIGS has proven useful in both reverse and forward genetic studies of plant metabolism, defense against viruses and pathogens, and development. Major advances in VIGS methodology include the introduction of TRV vectors [7,8] and the expansion of the number of VIGS hosts to include plants such as *Capsicum annuum *[9], *Solanum *species [10], *Papaver somniferum *[11], *Aquilegia vulgaris *[12], *Eschscholzia californica *[13] and Arabidopsis [14]. The advantages and disadvantages of VIGS have been reviewed [15-17].

A powerful application of VIGS is in fast-forward genetic screens, an approach first suggested by Baulcombe [18]. In such screens, cDNA libraries in VIGS vectors are used to infect a population of plants, with a different gene being silenced in each individual. No genetic mapping would be necessary to identify the disrupted gene as the cDNA fragment responsible for a given phenotype would be readily determined by sequencing the VIGS construct used to infect the plant in question. Lu *et al *[19] screened 4992 plant cDNAs in potato virus X (PVX) for their ability to suppress the hypersensitive response associated with Pto-mediated resistance against *Pseudomonas syringae*. High-throughput screens using tobacco mosaic virus (TMV) in *N. benthamiana *and barley stripe mosaic virus in barley have also been reported [20]. As the use of VIGS increases, and its virus-host repertoire expands, it is likely that fast-forward VIGS screens will become more widespread.

A major disadvantage of VIGS is that incomplete silencing yields plants that consist of a mosaic of silenced and non-silenced tissue. This effect, which has been observed with all VIGS host plants, is an impediment to the broad application of this technique. Host inserts may decrease the effectiveness of VIGS since inserts that interfere with viral spread will decrease the amount of silenced tissue. Clear guidelines for designing VIGS constructs do not exist. For example, what region and how much of an individual cDNA is optimal? Is the mixture of short and long cDNAs found in conventional oligo(dT) primed cDNA libraries useful for fast-forward genetic screens with VIGS? With the aim of constructing cDNA libraries directly in a TRV VIGS vector, we sought to determine the properties of cDNA inserts that would produce the most efficient silencing.

In this study we measured the effectiveness of VIGS in *Nicotiana benthamiana *by silencing the *phytoene desaturase *(*PDS*) gene using TRV [7,21]. Silencing *PDS*, which encodes an enzyme in carotenoid biosynthesis, results in white leaf tissue due to photobleaching [22]; it is a commonly used marker in VIGS experiments. We tested the effect that host insert length, position with respect to the full-length mRNA and inclusion of homopolymeric regions (i.e. poly(A) tails) have on VIGS efficiency. We developed a method for construction of subtracted VIGS-cDNA libraries with optimal insert properties and describe its application to the construction of root-specific VIGS-cDNA libraries from *N. benthamiana*.

## Results

### Construction of TRV-*NbPDS *plasmids and determination of chlorophyll a levels in silenced plants

**Figure 1:**
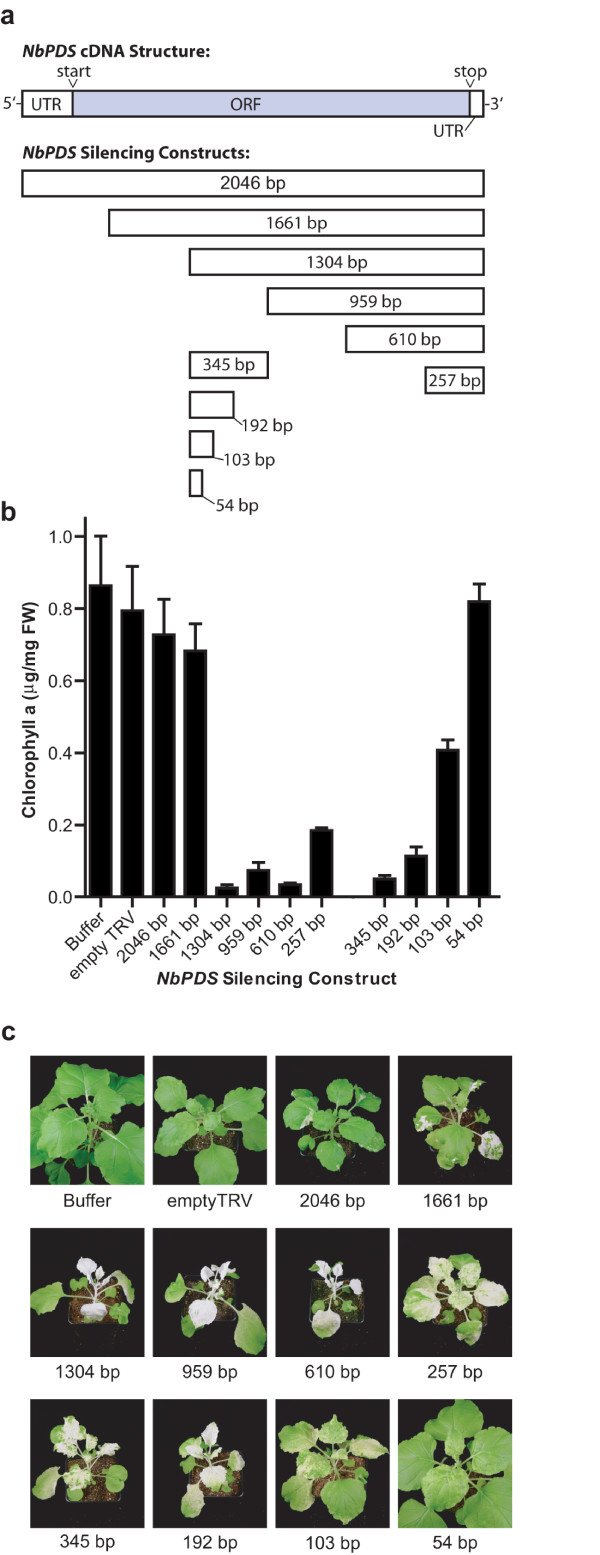
**Effect of cDNA insert length on the silencing of *PDS *in *N. benthamiana *leaves**. (a) Position and length of *NbPDS *cDNA fragments used for silencing relative to the full-length *NbPDS *cDNA. All inserts were in the antisense orientation relative to the TRV coat protein. (b) Chlorophyll a levels in the aerial parts of plants infected with TRV-*NbPDS *constructs. Buffer control plants received infiltration buffer only and empty TRV plants were infected with TRV lacking a silencing insert. Bars represent mean ± SD (n = 3). (c) Photographs of representative plants from TRV infected plants showing the extent of photobleaching.

A full-length cDNA clone of *N. benthamiana PDS *(*NbPDS*) was obtained using RACE PCR. The *NbPDS *cDNA was 2046 bp in length corresponding to a 1758 bp coding region, 223 bp 5' untranslated region (UTR) and a 65 bp 3' UTR (Figure 1a). The open reading frame of *NbPDS *showed high nucleotide identity with tobacco *PDS *(97%) and tomato *PDS *(90%). Our strategy for producing TRV-*NbPDS *constructs was to amplify fragments of *NbPDS *by PCR, clone them into a Gateway entry vector and recombine them into the TRV-RNA2 vector pYL279 [21]. The sequences of oligonucleotide primers used in this study and the primer combinations for amplifying VIGS inserts are shown in Table 1 and Table 2, respectively. All *NbPDS *cDNA inserts were in the antisense orientation relative to the TRV coat protein. Since all cloning procedures herein are performed with TRV cDNAs, in this paper we refer to the inserts as cDNAs. Following agro-infiltration and transcription driven by the 35S-promoter, the inserts exert their silencing effects as part of the single-stranded RNA genome of TRV. Four-week-old *N. benthamiana *plants were infiltrated with TRV-*NbPDS *constructs and TRV-RNA1. Plants that received infiltration buffer and those infected with a TRV-RNA2 vector lacking an insert served as negative controls. Control plants did not exhibit bleaching symptoms in any of our experiments although infection with empty TRV and TRV-*GFP *produced slightly stunted plants with some crinkled leaves.

**Table 1:** Sequences of oligonucleotides used for amplifying *NbPDS *inserts

**Primer name**	**Sequence (5'-3')**
NbPDSfor1	CACCGTTCAGGGGTATCTTTTTGTGG
NbPDSfor2	CACCAGAATTCGTAGTCCCAGTGC
NbPDSfor3	CACCATGCAGAACCTGTTTGGAGAA
NbPDSfor4	CACCTTCATAAACCCTGACGAGCTTT
NbPDSfor5	CACCTGGGAGTTCCTGTGATAAATG
NbPDSfor6	CACCCCCTTGCAAAGATCCCCTA
NbPDSrev1	GTGTACAACGCTAATTCAGCG
NbPDSrev2	GTTAAGTGCCTTTGACATGGC
NbPDSrev3	GACTTTCTCGGGGCCACGTAAG
NbPDSrev4	AGCGGCTGAACTCCCCTGGCT
NbPDSrev5	TTCCTTCCACTGCAACCGATC
NbPDSrev6	AAACTTATGCCCCATGGAGTC
NbPDSrev7	ATTTGGGTAAGCCCCAAAGAAT
NbPDSrev8	CTAGCTTCTCCAACTTTTGGAA
NbPDSrev9	CCGACAGGGTTCACAACCTGG
NbPDSrev10	(TTT)_8_V
NbPDSrev11	(TTT)_8_GTTAAGTGCCTTTGAC
NbPDSrev12	(CCC)_8_GTTAAGTGCCTTTGAC

^1 ^V represents A, C or G

**Table 2:** Oligonucleotide combinations used for amplification of *NbPDS *VIGS inserts

**Construct name**	**Primer combination**
NbPDS (2046 bp)	NbPDSfor1 × NbPDSrev1
NbPDS (1661 bp)	NbPDSfor2 × NbPDSrev1
NbPDS (1304 bp)	NbPDSfor3 × NbPDSrev1
NbPDS (959 bp)	NbPDSfor4 × NbPDSrev1
NbPDS (610 bp)	NbPDSfor5 × NbPDSrev1
NbPDS (257 bp)	NbPDSfor6 × NbPDSrev1
NbPDS (345 bp)	NbPDSfor3 × NbPDSrev2
NbPDS (192 bp)	NbPDSfor7 × NbPDSrev3
NbPDS (103 bp)	NbPDSfor7 × NbPDSrev4
NbPDS (54 bp)	NbPDSfor8 × NbPDSrev5
NbPDS (385 bp)	NbPDSfor1 × NbPDSrev6
NbPDS (357 bp)	NbPDSfor2 × NbPDSrev7
NbPDS (349 bp)	NbPDSfor4 × NbPDSrev8
NbPDS (353 bp)	NbPDSfor5 × NbPDSrev9
NbPDS (2046 bp + polyA)	NbPDSfor1 × NbPDSrev6
NbPDS (1661 bp + polyA)	NbPDSfor2 × NbPDSrev6
NbPDS (1304 bp + polyA)	NbPDSfor3 × NbPDSrev6
NbPDS (959 bp + polyA)	NbPDSfor4 × NbPDSrev6
NbPDS (610 bp + polyA)	NbPDSfor5 × NbPDSrev6
NbPDS (257 bp + polyA)	NbPDSfor6 × NbPDSrev6
NbPDS (345 bp + polyA)	NbPDSfor7 × NbPDSrev7
NbPDS (345 bp + polyG)	NbPDSfor7 × NbPDSrev8

The effect of silencing *PDS *was determined by analysis of chlorophyll a levels in pooled leaf and petiole tissue sampled from above the infiltrated leaves, with the reduction in pigment content through photobleaching used as a measure of silencing efficiency. In the case of *PDS *silencing, leaves may be green, white, pale yellow, pale green, or white with regions of dark green tissue. Since we did not separate green (non-silenced, unbleached) from white (silenced, bleached) tissue, measuring chlorophyll a levels summed the ability of the TRV construct to both spread within the plant and to silence *PDS *within infected tissue.

### Effect of cDNA size on silencing

TRV-*NbPDS *constructs containing full-length *NbPDS *(2046 bp) or *NbPDS *cDNAs truncated at the 5' end (1661 bp, 1304 bp, 959 bp, 610 bp and 257 bp) were amplified with gene specific primers. Figure 1a shows a schematic summary of *NbPDS *constructs used in this experiment. The length of the *NbPDS *insert influenced silencing efficiency as demonstrated by quantification of chlorophyll a and visual observation of leaf bleaching (Figure 1b and 1c). TRV-*NbPDS *(2046 bp) infected plants showed limited bleaching, with small regions of white tissue occasionally forming near major leaf veins, and chlorophyll a levels were only slightly lower than mock or empty TRV infected plants. Silencing efficiency increased in plants infected with TRV-*NbPDS *(1661 bp), although chlorophyll a levels were about 86% of empty TRV control levels and much leaf tissue remained green. Further increases in bleaching were found with the 1304 bp, 959 bp and 610 bp inserts, all of which produced plants with completely white leaves. TRV-*NbPDS *(257 bp), the shortest insert in this series, was a less effective silencer than the longer inserts and produced leaves that were mottled and pale green.

To further probe the effect of insert size on silencing, TRV-*NbPDS *constructs with inserts of 345 bp, 192 bp, 103 bp and 54 bp from the middle of the *NbPDS *cDNA were generated. TRV-*NbPDS *(345 bp) and TRV-*NbPDS *(192 bp) were capable of silencing *PDS *expression. However the TRV constructs with 103 bp and 54 bp inserts did not lead to effective silencing; indeed plants infected with TRV-*NbPDS *(54 bp) had chlorophyll levels that were slightly higher than controls. The bleached leaves formed by silencing with the 345 bp, 192 bp and 103 bp inserts all contained small regions of green tissue near leaf veins, giving them a mottled appearance (Figure 1c).

### Effect of cDNA position of PDS silencing

**Figure 2:**
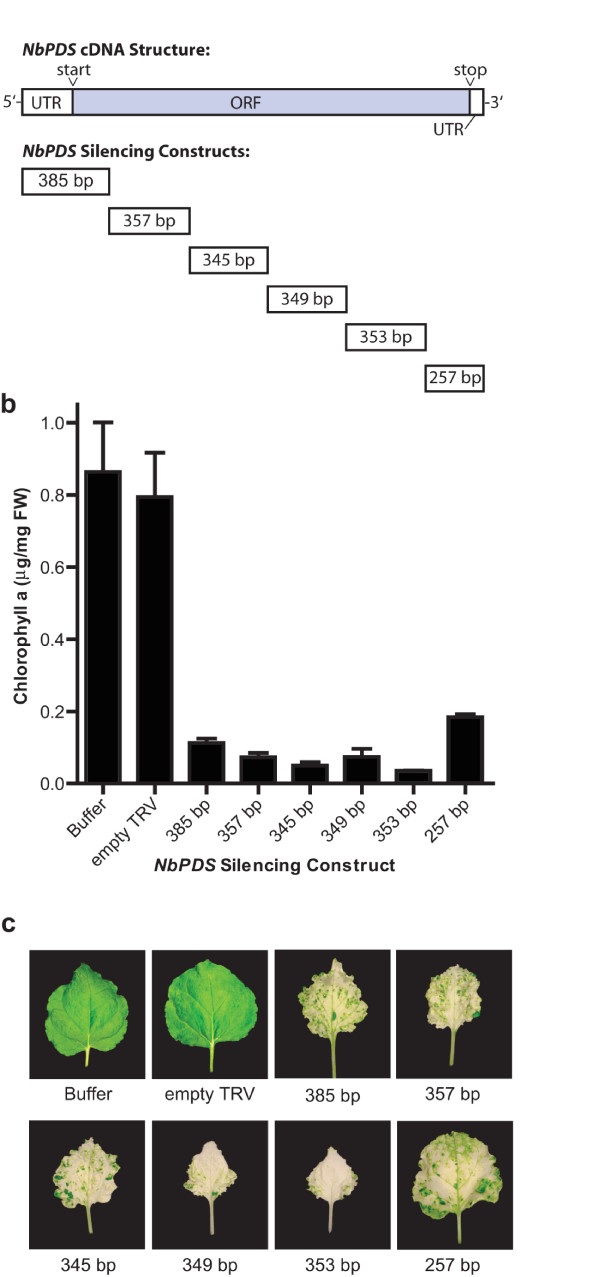
**Effect of cDNA insert position on the silencing of *PDS *in *N. benthamiana *leaves**. (a) Position and length of *NbPDS *cDNA fragments used for silencing relative to the full-length *NbPDS *cDNA. All inserts were in the antisense orientation relative to the TRV coat protein. (b) Chlorophyll a levels in the aerial parts of plants infected with TRV-*NbPDS *constructs. Buffer control plants received infiltration buffer only and empty TRV plants were infected with TRV lacking a silencing insert. Chlorophyll a values for buffer, empty TRV and some constructs are also shown in Figure 1B. Bars represent mean ± SD (n = 3). (c) Photographs of representative leaves from TRV infected plants showing the extent of the photobleaching.

We next tested if the position of the insert with respect to the full-length cDNA affected silencing. TRV constructs were synthesized using primers designed to amplify a non-overlapping series of short *NbPDS *fragments (Figure 2a). In this manner we synthesized constructs of TRV-*NbPDS *1–385 (385 bp), TRV-*NbPDS *386–742 (357 bp), TRV-*NbPDS *743–1087 (345 bp), TRV-*NbPDS *1088–1436 (349 bp) and TRV-*NbPDS *1437–1789 (353 bp), which together with the *NbPDS *1788–2046 (257 bp) construct, gave complete coverage of *NbPDS*. Plants infected with these constructs showed that all were effective in silencing but slightly more bleaching was produced by constructs located in the middle of the cDNA compared to the 5' and 3' ends (Figure 2b). This effect was apparent in the fine green mottling on the leaves of TRV-*NbPDS *1–385 (385 bp) and TRV-*NbPDS *1788–2046 (257 bp) infected plants while silencing inserts from the middle of the *NbPDS *cDNA gave more uniformly bleached leaves.

### Effect of homopolymeric regions on PDS silencing

**Figure 3:**
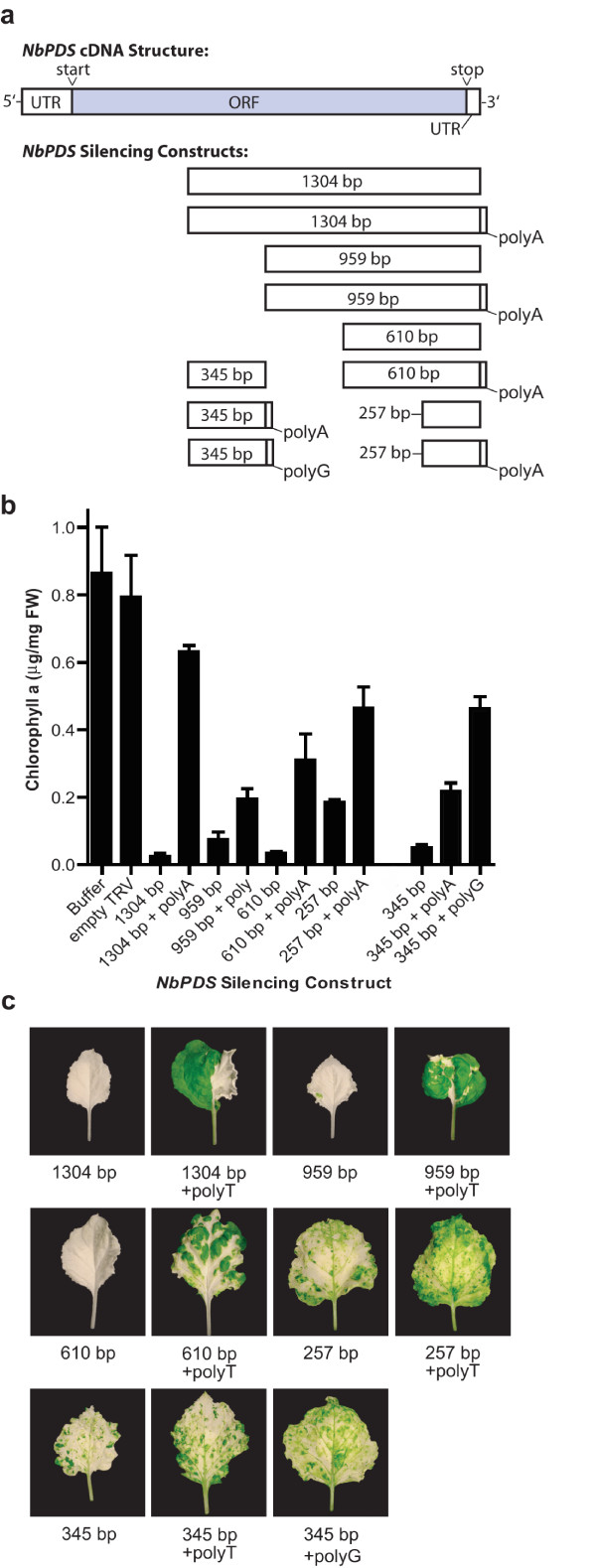
**Effect of a 24 bp homopolymeric region on the silencing of *PDS *in *N. benthamiana *leaves**. (a) Position and length of *NbPDS *cDNA fragments used for silencing relative to the full-length *NbPDS *cDNA. All inserts were in the antisense orientation relative to the TRV coat protein. (b) Chlorophyll a levels in the aerial parts of plants infected with TRV-*NbPDS *constructs. Buffer control plants received infiltration buffer only and empty TRV plants were infected with TRV lacking a silencing insert. Chlorophyll a values for buffer, empty TRV and some constructs are also shown in Figure 1B and 2B. Bars represent mean ± SD (n = 3). (c) Photographs of representative leaves from TRV infected plants showing the extent of the photobleaching. Leaves from 345 bp and 257 bp constructs are also shown in Figure 2C.

Since cDNAs often contain 3' homopolymeric regions (i.e. poly(A) tails) that are incorporated as part of the cDNA synthesis process, we tested the effect that inclusion of homopolymeric regions has on VIGS efficiency. Four silencing inserts that had been shown to be effective silencers in our first experiment (1304 bp, 959 bp, 610 bp, 257 bp) were amplified using an oligo(dT) primer that added a 3' 24 bp homopolymeric region. This gave inserts of 1328 bp, 983 bp, 634 bp and 281 bp. Plants infected with TRV constructs containing polyadenylated inserts showed reduced silencing efficiency compared to constructs lacking this region (Figure 3). The inclusion of the 24 bp poly(A/T) region had a particularly dramatic effect on the silencing effectiveness of the 1304 bp and 257 bp inserts. Silencing with the 1304 bp insert led to greater than 95% reduction in chlorophyll a levels compared to empty TRV control plants while a 21% decrease was found with the 1328 bp homopolymeric insert. We also added a poly(G) region to the 345 bp insert. The silencing effectiveness of this insert, which gave a 94% reduction in chlorophyll a levels compared with empty virus controls, was strongly reduced by the addition of the poly(G) sequence (Figure 3b).

### Construction of subtractive VIGS-cDNA libraries

**Figure 4:**
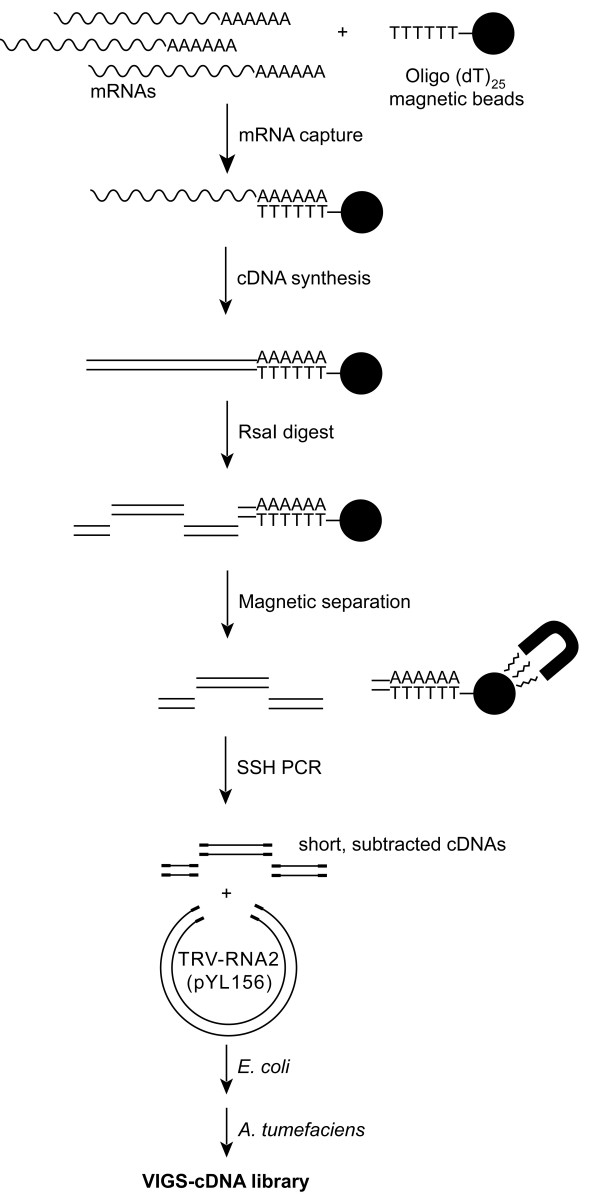
**Schematic representation of VIGS-cDNA library synthesis method**. Magnetic bead linked oligo(dT) primers are used to immobilize mRNAs and prime cDNA synthesis. RsaI digestion yielded short (~600 bp) fragments lacking 3' homopolymeric regions. Suppression subtractive hybridization (SSH) was used to subtract cDNA fragments present in a driver population of cDNAs from those present in a tester population [23, 24]. After ligation into a TRV-RNA2 vector, and amplification in *E. coli*, the cDNA inserts are transformed into *A. tumefaciens *to produce a collection of cDNAs suitable for both VIGS and EST sequencing.

Based on the optimal properties for VIGS inserts defined by the above experiments, we developed a cDNA library synthesis method suitable for constructing VIGS libraries. This method, which is outlined in Figure 4, utilizes suppression subtractive hybridization (SSH) as a means to produce subtracted libraries of short cDNAs that differ in their expression between a reference tissue (driver) and one with an altered gene expression profile (tester) [23]. We used magnetic bead-linked oligo(dT) primers to capture mRNA and then synthesized cDNA on this solid-phase support. Subsequent digestion of the cDNA with RsaI freed the cDNA in ~600 bp fragments leaving the 3' ends and their poly(A/T) regions immobilized on the magnetic beads. Subsequent SSH cloning and ligation procedures were performed as described by Diatchenko *et al. *[24]. In order to introduce cDNA fragments into the TRV-RNA2 VIGS vector, we modified the final PCR amplification procedure by using primers that introduced EcoRI and BamHI recognition sites onto the ends of the subtracted amplicons. Subsequent digestion, ligation into the TRV-RNA2 VIGS vector pYL156 and transformation into *E. coli *gave a high-titre cDNA library in a plasmid vector. The library was amplified in *E. coli *before transformation into Agrobacterium.

To demonstrate the utility of the VIGS-cDNA library synthesis method, and with the objective of using VIGS to study alkaloid metabolism in the Solanaceae, we constructed two subtractive VIGS-cDNA libraries from *Nicotiana benthamiana *roots. Nicotine is synthesized in the roots of *Nicotiana *species and transported to the leaves where it serves as a defense against insect herbivory [25]. Nicotine biosynthesis is up-regulated in response to insect feeding, wounding or the application of jasmonates [26]. We treated the roots of hydroponically grown *N. benthamiana *plants with methyl jasmonate and harvested roots 1, 3, 7 and 10 hours after treatment. Quantitative real-time PCR was use to confirm that the expression several genes involved in nicotine biosynthesis, including ornithine decarboxylase, putrescine *N*-methyltransferase (*PMT*), and quinolinate phosphoribosyl transferase, increased with methyljasmonate treatment (data not shown). Two subtracted cDNA libraries were constructed: (1) NBREL1 using by mRNA pooled from methyljasmonate-treated roots as tester and untreated root mRNA as driver, and (2) NBLEL2, using by mRNA pooled from methyljasmonate-treated roots as tester and untreated leaf mRNA as driver. The libraries were amplified in *E. coli*, transformed into Agrobacterium and plated on agar. For each library, 1920 colonies were transferred to 96-well plates, grown overnight and used as templates for PCR. The resulting amplicons were sequenced from the 5' end. The Agrobacterium cultures were stored as glycerol stocks for use in VIGS experiments.

**Figure 5:**
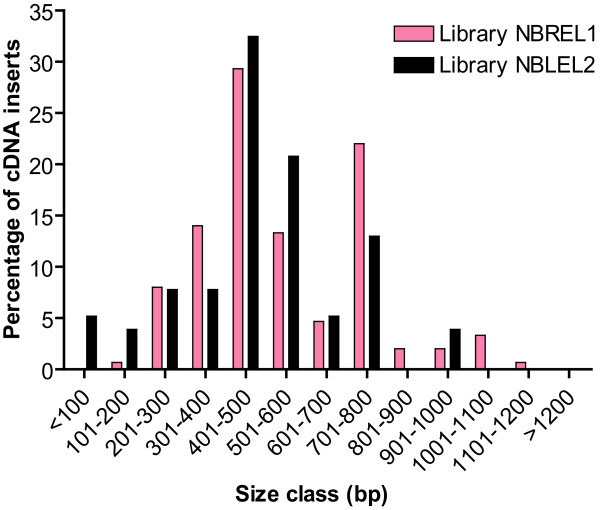
**Size distribution of cDNA inserts in two subtracted VIGS-cDNA libraries**. cDNA library NBREL1 was constructed using mRNA from methyljasmonate-treated *N. benthamiana *roots as tester and untreated root mRNA as driver. cDNA library NBLEL2 was constructed using by mRNA pooled from methyljasmonate-treated roots as tester and untreated leaf mRNA as driver. We analyzed 150 sequenced cDNAs from NBREL1 and 77 from NBLEL2 to determine the length of each insert.

The properties of the VIGS-cDNA libraries are shown in Table 3. The sequencing success rate of 77% was relatively low owing to variation in the amount of PCR products amplified from Agrobacterium colonies. Analysis of 150 cDNAs from NBREL1 and 77 from NBLEL2 showed that insert sizes ranged from 150 bp to 1150 bp with the majority (30%) in the size range of 401–500 bp (Figure 5). Less than 1% of NBREL1 and about 9% of NBLEL2 contained inserts smaller than 200 bp. We detected 18 cDNAs (0.5% of the total number sequenced) with poly(A) tails confirming that our strategy of immobilizing cDNAs before RsaI digestion was successful. One problem that was apparent from EST sequencing was that some inserts consisted of fragments of two or more genes. Such chimeric clones were of relatively low frequency (e.g. five of 150 cDNAs in NBREL1).

**Table 3:** Summary of VIGS-ESTs from methyljasmonate-treated roots of *N. benthamiana*

Number of ESTs for each cDNA library:	NBREL1	1920
	NBLEL2	1920
Total number of ESTs		3840
Number of high-quality ESTs^a^		2948
Average length of high-quality ESTs (bp)		290
Number of contigs		364
Number of singletons		1327
Number of putative unique transcripts^b^		1691

^a ^After removal of vector, low-quality, poly(A/T) and simple repeats.

^b ^Number of unique transcripts is the number of contigs and singletons.

### Silencing an enzyme of nicotine biosynthesis reduces leaf nicotine levels

**Figure 6:**
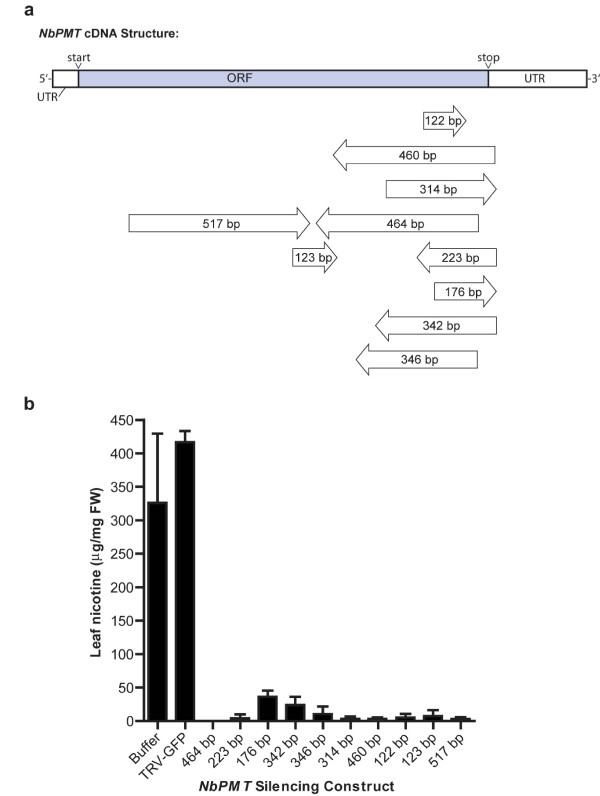
**Silencing of putrescine *N*-methyltransferase (*PMT*) gene using cDNAs derived from the VIGS-cDNA libraries, NBREL1 and NBLEL2**. (a) Position and length of *NbPMT *cDNA fragments of *NbPDS *used for silencing relative to the full-length *NbPMT *cDNA. Orientation of the inserts relative to the TRV coat protein is indicated by arrows. (b) Leaf nicotine levels in plants infected with TRV-*NbPMT *constructs as determined by HPLC. Buffer control plants received infiltration buffer only and TRV-*GFP *plants were infected with TRV containing a non-functional 363 bp *GFP *insert. Bars represent mean ± SD (n = 4).

To test the effectiveness of gene silencing using TRV constructs derived from the subtracted VIGS-cDNA library, we silenced *PMT*, a gene encoding a rate-limiting enzyme in the nicotine biosynthetic pathway. VIGS of *PMT *has previously been shown to reduce nicotine levels in *Nicotiana attenuata *[27]. Fifteen *PMT *ESTs, which corresponded to a fourteen-membered contig and one singleton, were present in the subtracted library and we selected ten representative constructs for VIGS. The ten inserts we distributed across the *PMT *cDNA but most aligned to the 3' end (Figure 6). They ranged in size from 122 bp to 517 bp and were in both the sense and antisense orientation relative to the TRV coat protein.

The TRV-*NbPMT *constructs were agro-infiltrated into three-week-old *N. benthamiana *plants. Control plants received infiltration buffer or a TRV-*GFP *construct. We used the latter because it reduced virus symptoms such as stunting and leaf curling compared with the empty TRV-RNA2 control. Plants were grown for three weeks and sprayed with a solution of methyljasmonate to stimulate nicotine production. After five days leaf nicotine levels were determined by reversed-phase ion-pair HPLC. Nicotine levels in buffer control of TRV-*GFP *infected plants were 326 μg/mg fresh weight (FW) and 416 μg/mg FW, respectively. Nicotine levels were reduced by greater than 90% in plants infected with all ten TRV-*NbPMT *constructs (Figure 5b). The levels of leaf nicotine in silenced plants ranged from undetectable to 37 μg/mg FW (mean of 10 μg/mg FW, n = 10). Both sense and antisense orientated inserts performed with equal efficiency.

## Discussion

A significant limitation of VIGS is the incomplete spread of virus or poor silencing in infected tissues. The resulting variegation complicates phenotypic analysis of the majority of genes that, unlike *PDS*, do not produce visible phenotypes. For this reason, optimizing the silencing efficiency of VIGS constructs is crucial for successful silencing experiments. We performed our experiments with TRV because it is the mostly widely used VIGS vector and chose to target *PDS *because it results in a visible phenotype and has been used in numerous VIGS studies. Since *PDS *is encoded by a single gene in plants, is expressed at low levels and, at 2.1 kb, is longer than average, it may not be representative of all plant mRNAs. In addition, *N. benthamiana *is more susceptible to virus infection than many other host plants, possibly due to the lack of a virus-inducible RNA-dependent RNA polymerase [28]. Despite these factors, which may limit the extension of our findings to the silencing of other genes and in other host plants, our *PDS *experiments provide a starting point for designing VIGS constructs.

### Effect of cDNA insert length and position on silencing

The influence of host insert size on VIGS efficiency has received little experimental attention. Our results show that cDNAs longer than about 1500 bp, such as the 1661 bp *NbPDS *insert, gave little or no silencing of *PDS*. One explanation for this result is that viral replication is impaired when a large foreign sequence is inserted in its genome. Another possibility is that large inserts are removed by recombination and that, although TRV is spreading systemically, the silencing insert has been lost. The replication and movement of RNA viruses is often impaired when foreign sequences are inserted into the viral genome and such sequences are readily deleted through recombination. Insertion of a 1.6 kb bacterial β-glucoronidase (*GUS*) gene in plum pox potyvirus delayed virus accumulation and led to deletion of *GUS *after passaging in *N. benthamiana *[29]. Similarly, recombinant poliovirus containing *GFP *(~750 bp) showed impaired replication and rapid deletion of the *GFP *gene in HeLa cells [30]. *GFP *and *GUS *tagging of lettuce mosaic virus attenuated virus symptoms [31]. Tobraviruses including TRV show a high degree of genome alteration through recombination [32], which suggests that non-optimal inserts may be quickly deleted or altered through recombination. Limitations on insert size due to packaging constraints have been observed for some viruses such as adeno-associated virus [33]. In designing the TRV vectors used in this study, Liu *et al. *[7,21] deleted genes encoding the 28.7 k and 32.8 k proteins from a wildtype Ppk20 virus and inserted multiple cloning and Gateway sites between the coat protein and the 3' untranslated region. This reduced the size of TRV-RNA2 from 3855 bp to 2103 bp, or about 1.7 kb, for pYL156 and 2.3 kb for pYL279. This is near the upper limit that we determined, and suggests that there may be natural constraints for the size of the TRV genome. The size constraints uncovered in our study likely only apply to TRV and not other VIGS viruses such as those based on geminiviruses. Further experiments will be needed to determine the optimal inserts properties in other VIGS vectors.

The lower limit for efficient *PDS *silencing using TRV was determined to be about 190 bp and 122 bp for *NbPMT*. The difference in these lengths makes it clear that there is variation in the silencing of different genes. However, the fact that *PDS *required a ~200 bp fragment suggests that other researchers should use this as a minimum length rather than the 122 bp observed with *PMT*. The lower limit sizes (190 and 122 bp) are larger than the 23 nucleotides that was obtained using potato virus X (PVX), although such short fragments gave patchy phenotypes [34]. This discrepancy may be due to the use of different VIGS viruses (PVX vs. TRV) in the experiments. In addition, the PVX study used a *GFP *transgene, rather than an endogenous gene, as a silencing target. Inserts longer than 190 bp likely lead to the generation of more and diverse siRNAs, and these silence more effectively than short inserts.

The position of the VIGS fragment relative to the full-length cDNA had a small effect on silencing efficiency (Figure 2). The 385 bp 5' fragment and the 3' 257 bp fragment, both of which included both coding sequence and untranslated regions, showed increased chlorophyll a levels compared to the other four constructs tested. The 257 bp insert from the extreme 3' end in particular was less effective. Using PVX, Ruiz *et al. *[35] assayed *PDS *silencing in *N. benthamiana *and found that inserts derived from the middle of the *NbPDS *cDNA (415 bp and 212 bp fragments), at the 5' end (377 bp fragment) were able to silence *PDS*. Inserts targeting 5' and 3' located introns (167 bp and 223 bp, respectively) did not trigger silencing. In general, other papers have recommended situating the VIGS fragment in the middle of the coding region of the transcript, except were targeting the 5' or 3' ends gives more specific silencing of gene family members (e.g. [36]). Our study indicates almost any part of the cDNA can be used to trigger silencing but that avoiding the extreme 5' and 3' ends may increase silencing efficiency.

### Effect of homopolymeric regions on silencing

We observed that the presence of a 24 bp poly(A) region dramatically reduced the efficiency of silencing (Figure 3). A possible explanation for this effect is that poly(A) binding proteins interacting with this region interfere with viral replication. However, addition of both poly(A) and poly(G) sequences to the 345 bp insert, which previous experiments had shown acts as an efficient silencer, compromised silencing. These data indicate the effect was not specific to polyadenylated regions but rather homopolymeric regions in general. The TRV genome does not naturally contain polyadenylated regions [32].

### cDNA libraries for VIGS

Expressed sequence tags (ESTs) have proven to be one of the most useful types of genomic information, especially in eukaryotic organisms with large, unsequenced genomes. By constructing cDNA libraries directly in a VIGS vector and then sequencing randomly selected cDNAs, we aimed to produce a collection of VIGS-ready cDNAs in Agrobacterium and a corresponding EST dataset that could be used for functional genomics. The cDNA library method we developed produces short cDNA inserts lacking poly(A/T) regions. In addition, the use of subtractive cDNA library methodology allows for the enrichment of clones from certain tissues or those expressed in response to treatments (e.g. methyljasmonate treated *N. benthamiana *roots). Using this method we constructed two subtractive VIGS-cDNA libraries, analyzed their corresponding EST datasets for cDNAs matching a key enzyme in the nicotine pathway, and demonstrated efficient silencing using ten different TRV-*NbPMT *constructs. We are currently applying this approach to identify additional enzymes and regulatory proteins involved in alkaloid metabolism in the Solanaceae. This VIGS-EST approach should be useful for both fast forward and reverse genetics experiments in plants for which genomic information is limited.

## Conclusion

Based on our experiments with *NbPDS *and *NbPMT*, we put forward the following guidelines for designing effective VIGS constructs in TRV vectors: (1) Insert lengths should be in the range of ~200 bp to ~1300 bp, (2) they should be positioned in the middle of the cDNA and (3) homopolymeric regions should not be included in the silencing insert. Although these properties were determined using only two genes in an optimal VIGS host, *N. benthamiana*, they should be useful for increasing the efficiency of VIGS in other plant hosts.

## Methods

### Isolation of *Nicotiana benthamiana *full-length PDS cDNA and construction of TRV plasmids

A full-length *NbPDS *cDNA was obtained by RACE-PCR (GeneRacer kit, Invitrogen) using gene-specific primers based on a published *NbPDS *cDNA fragment. The full-length cDNA was amplified from RACE-ready cDNA with primers corresponding to the 5' end of the *NbPDS *cDNA and the GeneRacer 3' nested primer using Pfu polymerase and sequenced. *NbPDS *cDNA fragments from *N. benthamiana *were PCR amplified with Taq polymerase using the oligonucleotide primers shown in Table 1 and the full-length *NbPDS *cDNA as template. Due to errors in the draft *NbPDS *sequence, two primers (NbPDSfor2 and NbPDSrev6) had sequences that differed from the final sequence. PCR products were gel purified and ligated into pENTR-D-TOPO (Invitrogen). The TRV-*GFP *construct contained a non-functional 363 bp fragment of *GFP*. After sequencing, cDNA inserts were recombined into the Gateway TRV-RNA2 vector pYL279 [21] using LR recombinase (Invitrogen). Constructs were confirmed by sequencing before transformation into *A. tumefaciens *C58 via electroporation. The nucleotide sequences of *NbPDS *[GenBank: EU165355] and *NbPMT *[GenBank: EU165356] have been deposited in GenBank.

### Growth and infiltration of *Nicotiana benthamiana *plants

*N. benthamiana *plants were grown in soil in a controlled environment chamber with 16 hour/23° days and 8 hour/20° nights at 100 μmol/m^2^/s light intensity. Cultures of *A. tumefaciens *containing TRV-RNA1 or TRV-RNA2 plasmid were cultured separately overnight at 28°C. After centrifugation, bacterial cell pellets were resuspended in infiltration buffer containing 1 mM MES (pH 5), 10 mM MgCl_2 _and 100 μM acetosyringone to OD_600 _= 1 and allowed to stand at room temperature for 3–6 hours before infiltration. Suspensions of TRV-RNA1 and TRV-RNA2 constructs were mixed 1:1 and infiltrated into the underside of the upper leaves of 3–4 week old plants using a 1 ml syringe. Mock infected plants received infiltration buffer.

### Chlorophyll a analysis

*N. benthamiana *leaves and petioles were sampled 3–4 weeks after infiltration, frozen in liquid nitrogen and ground to a fine powder. Portions of approximately 150 mg were transferred to a microcentrifuge tube and accurately weighed. One-ml of 90% (v/v) aqueous acetone was added and the sample vortexed for 1 min. The extract was centrifuged for 1 min at 13,200 g and aliquots of the supernatant measured at 630, 645, 663 and 750 nm using a UV-vis spectrophotometer (Lambda 35, PerkinElmer) and chlorophyll a amounts calculated [37]. Values were calculated as mean and standard deviation for three plants (n = 3) of one infiltration experiment. Representative plants or leaves were photographed at the time of chlorophyll a analysis using a digital camera.

### Construction of subtracted VIGS-cDNA libraries

*N. benthamiana *seedlings were grown hydroponically in 0.25× Hoagland's solution supplemented with iron chelate solution and oxygenated using an aquarium bubbler. Roots from three-week old plants were sampled immediately before and at 1, 4, and 7 h after addition of methyljasmonate to a final concentration of 11 μM. Total RNA was isolated from 450 mg each of untreated leaves, untreated roots, and a combined root sample composed of 150 mg roots each from the 1, 4 and 7 h time points using a RNeasy midi kit (Qiagen). A PCR-select subtractive cDNA library kit (Clontech) was used for cDNA synthesis with some modifications. Briefly, about 250 μg of total RNA was mixed with 300 μl of Oligo(dT)_25 _Dynabeads (Dynal Biotech) in binding buffer (20 mM Tris-HCl pH 7.5, 1 M LiCl, 2 mM EDTA). After a 10 min incubation, the beads were washed three times with washing buffer B (10 mM Tris-HCl pH 7.5, 0.15 M LiCl, 1 mM EDTA), followed by washing twice with first strand buffer. The washed beads containing mRNA were resuspended in 40 μl of cDNA synthesis cocktail (8 μl 5× first strand buffer, 4 μl 10 mM dNTPs, 24 μl RNase-free water and 4 μl (8 U) AMV reverse transcriptase) and incubated at 42°C for 1.5 h. The second strand synthesis was completed by addition of 120 μl of second strand synthesis cocktail (32 μl of 5× second strand buffer, 3.2 μl of 10 mM dNTPs, 8 μl of 20× enzyme cocktail and 76.8 μl RNase free water) and incubation at 16°C for 2 h, followed by addition of 4 μl (12 U) T4 DNA polymerase and incubation for 30 min. The reaction was stopped by addition of 20 μl 0.5 M EDTA. The beads were magnetically separated, the supernatant removed, the beads resuspended in 500 μl of wash buffer (5 mM Tris-HCl pH 7.5, 0.5 mM EDTA, 1 M NaCl, 1% SDS and 10 μg/ml glycogen) and heated at 75°C for 15 min. The beads were then washed three times with wash buffer (5 mM Tris-HCl pH7.5, 0.5 mM EDTA, 1 M NaCl and 200 μg/ml BSA), followed by two more washes with RsaI buffer. The beads were resuspended in 84 μl water, 10 μl 10× RsaI buffer, 3 μl (30 U) RsaI, and incubated at 37°C overnight. The freed cDNA was isolated by magnetic separation of the beads and was used for adapter ligation, hybridizations and primary PCR as described in the manufacturer's protocol (Clontech). Secondary PCR was performed using primers 5'-CGGGATCCTCGAGCGGCCGCCCGGGCAGGT-3' (BamHI site underlined) and 5'-CGGAATTCAGCGTGGTCGCGGCCGAGGT-3' (EcoRI site underlined). The PCR-select amplified cDNA fragments (700 ng) were digested with EcoRI and BamHI, followed by ligation into a similarly digested TRV-RNA2 vector, pYL156. The ligation mixture was electroporated into DH10B *E. coli *competent cells to give a primary library of 9 × 10^5 ^cfu. The library was amplified on agar plates, plasmid DNA isolated and used to transform *A. tumefaciens *C58 via electroporation. The ligation efficiency as determined by colony PCR using vector primers 5'-GTTACTCAAGGAAGCACGATGAG-3' and 5'-CAGTCGAGAATGTCAATCTCGTAG-3' was 98%. Colonies were transferred to 96-well plates containing 100 μl of LB medium containing 50 μg/ml kanamycin and 10 μg/ml rifampicin. To amplify cDNA inserts for sequencing, PCR was performed using the above vector primers and 1 μl of Agrobacterium culture as template. The resulting PCR products were sequenced directly using BigDye terminators and the primer 5'-GTTACTCAAGGAAGCACGATGAG-3'.

### Nicotine analysis by high-performance liquid chromatography

Three weeks after agro-infiltration, the leaves of *N. benthamiana *plants were sprayed with a solution of 0.1% (v/v) of methyljasmonate in water containing 0.1% Tween-20. Five days after spraying, three leaf disks (~30 mg) were collected from the upper leaves directly into a 2 ml screw top vial. The tubes were briefly centrifuged to pellet the leaf disks and 50 μl of 1 mm zirconium beads (BioSpec Products) and 100 μL of 50 mM citrate buffer (pH 3)-methanol (70:30) were added. The tubes were homogenized for three minutes in a tissue disrupter (Mini-Beadbeater-96, BioSpec Products), followed by sonication for 10 minutes in an ultrasonic bath. The homogenate was centrifuged at 4° for five minutes at 14,000 rpm in a microcentrifuge and the supernatant transferred to a 96-well filter plate (0.45 μm, MultiScreen Solvinert, Millipore). The plate was centrifuged at 4°C for 2 min at 4,000 rpm. The filtrate was transferred to an autosampler vial and a 20 μl aliquot analyzed by HPLC. HPLC analysis was performed on a Waters 2695 separations module equipped with a Waters XTerra C18 reversed phase column (4.6 × 150 mm, 5 μm) with precolumn at a column temperature of 60°C. The mobile phase consisted of 50 mM citrate buffer (pH adjusted to 3.0 with triethylamine) containing 10 mM octanesulfonic acid-MeOH (76:24). The separation was performed for 15 min at a flow rate of 1 ml/min. Nicotine was detected at 261 nm via photodiode array detection. Quantification was performed using peak area by comparison to a standard curve (*r*^2 ^0.999) derived from injection of nicotine solutions ranging in concentration from 1 μg/ml to 1040 μg/ml.

## Competing interests

The author(s) declare that they have no competing interests.

## Authors' contributions

EW carried out the cloning procedures, constructed the cDNA libraries, analyzed chlorophyll levels and helped draft the manuscript. JP conceived of the study, and participated in its design and coordination, grew plants, performed nicotine analysis and helped to draft the manuscript. All authors read and approved the final manuscript.
